# A Double-edged Sword: Uric Acid and Neurological Disorders

**DOI:** 10.4172/2168-975X.1000109

**Published:** 2013-11-01

**Authors:** Pu Fang, Xinyuan Li, Jin Jun Luo, Hong Wang, Xiao-feng Yang

**Affiliations:** 1 Center for Metabolic Disease Research, Temple University School of Medicine, Philadelphia PA, 19140, USA; 2 Center for Cardiovascular Research, Temple University School of Medicine, Philadelphia PA, 19140, USA; 3 Sol Sherry Thrombosis, Temple University School of Medicine, Philadelphia PA, 19140, USA; 4 Department of Pharmacology, Temple University School of Medicine, Philadelphia PA, 19140, USA; 5 Department of Neurology, Temple University School of Medicine, Philadelphia PA, 19140, USA

**Keywords:** Uric acid, Hyperuricemia, Neurological disorders, Caspase-1 activation, Inflammation

## Abstract

Uric Acid (UA), historically considered as a waste of cellular metabolism, has now received increasing attention because it was found to directly participate in the pathogenesis of many human diseases including neurological disorders. On one hand, low levels of UA are detrimental to the neurons because of its induction it impairs antioxidant capacity in the cell. High levels of UA, on the other hand, lead to an inflammatory response contributing to gout or neuroprotection. In this review, we summarize this biphasic function of uric acid and highlight potential therapeutic targets to treat UA-related neurological diseases.

## Introduction

Despite great progress in understanding the molecular mechanisms underlying human neurodegenerative diseases, the prevalence of nervous system disorders increases, including Alzheimer’s disease (AD), Huntington’s disease (HD), Parkinson’s disease (PD), and Multiple Sclerosis (MS). Uric acid (UA) is a particularly interesting molecule that may be involved in the pathogenesis of AD, HD, PD, and MS [1–4]. AD is the most common cause of dementia affecting estimated 24 million people worldwide [5] due to loss of neurons and synapses in the cerebral cortical and certain subcortical regions. HD is an inherited neurodegenerative disorder in the basal ganglia affecting muscle coordination and leading to cognitive decline and psychiatric symptoms. PD results from the dopamine neuronal degeneration in the substantia nigra in the midbrain, causing resting tremor, rigidity, bradykinesia, posture and ambulatiing difficulty. AD, HD and PD are progressively neurodegenerative disorders with unknown etiologies; while MS is an autoimmune-mediated inflammatory disorder causing central nerve system demyelination. Currently there is no cure for these diseases.

## Discussion

UA is the end product of purine metabolism. The formula of UA is C_5_H_4_N_4_O_3_ (7,9-dihydro-1H-purine-2,6,8(3H)-trione). It has a molecular weight of 168 Daltons. UA is converted from two nucleotides, adenosine monophosphate and guanine monophosphate. Adenosine monophosphate is converted to inosine by two different mechanisms; either initially removing an amino group by deaminase to form Inosine Monophosphate (IMP) followed by dephosphorylation by a nucleotidase to form inosine, or initially removing a phosphate group by a nucleotidase to form adenosine followed by deamination to form inosine. Guanine monophosphate is converted to guanosine by nucleotidase. Then inosine and guanosine are further converted to hypoxanthine and guanine, respectively, by Purine Nucleoside Phosphorylase (PNP). Both hypoxanthine and guanine form xanthine, and then xanthine is oxidized to form UA. In physiologic conditions, UA is excreted in urine.

High serum levels of UA, or hyperuricemia, is defined as a metabolic pathology with a blood UA concentration greater than 7.0 mg/dL in men and 6.0 mg/dL in women. Renal disease is one of the causes leading to hyperuricemia. Babies often have hyperuricemia because they are born with nephrons fewer than healthy adults [6]. In addition, hyperuricemia can result from increased UA production such as diets rich in purine or fructose, exposure to lead, and adverse effects from medical intervention, e.g. chemotherapy for leukemia [7]. Deficiency of enzymes resulting from genetic mutations may also contribute to increased blood UA levels. Both Hypoxanthine-Guanine Phosphoribosyl Transferase (HGPRT) and glucose-6-phosphatase deficiencies can cause accumulation of 5-Phosphoribosyl-Alpha-Pyrophosphate (PRPP), which is used in the salvage pathway of hypoxanthine, xanthine and guanine, adversely leading to hyperuricemia.

It is well known that hyperuricemia is the major etiology of gout in adults. Gout is an inflammatory medical condition characterized by painful, red, tender, hot, and swollen joints, which is caused by the deposition of Monosodium Urate (MSU, UA crystal) in the joints, tendon, kidney and other tissues. The deposition subsequently activates caspase-1/inflammasome complex and increases the secretion of caspase-1 substrates including proinflammatory cytokines interleukin-1β (IL-1β) and IL-18 [8–10], an important intracellular mechanism triggering the cascade for inflammation. Lesch-Nyhan syndrome is a rare X-linked inherited disorder caused by the deficiency of HGPRT resulting in hyperuricemia and hyperuricosuria. Lesch-Nyhan syndrome is associated with severe gout and renal insufficiency from early life. Its neurological signs include poor muscle control, moderate mental retardation and self-mutilating behavior with facial grimacing, involuntary writhing, and repetitive movements of the arms and legs similar to those seen in HD. In addition to gout and Lesch-Nyhan syndrome, hyperuricemia may potentiate cardiovascular disorders and stroke [8].

**Figure 1:**
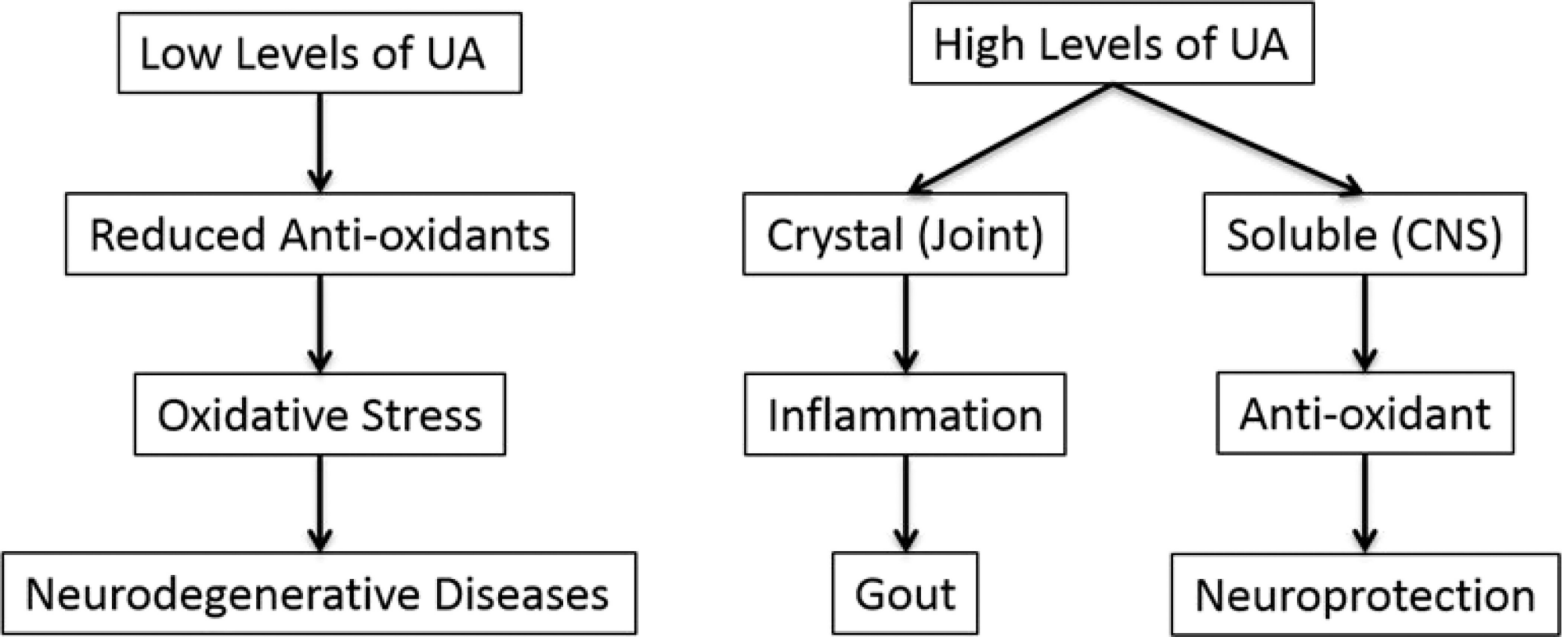
UA’s effect is organ-dependent, plasma level dependent and soluble/crystal status dependent. UA-uric acid; CNS-central nervous system

While hyperuricemia has been linked with gout, clinical observations disclosed reduced serum UA levels in some neurological diseases [11–16] (Figure 1) including AD, HD, PD, and MS [1–4]. Thus, it has been argued whether the lower UA levels are relevant to neurodegeneration while the hyperuricemic levels may have a neuroprotective action. In a large population-based cohort study conducted in Canada, De Vera et al. confirmed a negative relationship between gout and PD on patients whose ages were greater than 65 [14]. Using the British Columbia Linked Health Database and PharmaCare data, the incidence rates of PD in 11,258 gout patients were compared with 56,199 matched controls. Over an 8-year median follow up, there was a 30% reduction in the risk of developing PD in both male and female patients with a history of gout, independent of age, sex, prior comorbid conditions, nonsteroidal anti-inflammatory drugs, and diuretic use. A similar trend has been shown in a recent study exploring the relationship between UA levels and HD [13]. By performing a secondary analysis of the HD CARE-HD trial, Auinger et al. demonstrated slower HD progression with higher UA levels. Additionally, lower UA levels have been observed in AD and MS patients [2,13], suggesting that UA may play a role in prevention of neurodegeneration [17].

Several studies have suggested a causal role of UA in the neurodegenerative diseases. The possible neuroprotective role of UA has been shown by its anti-oxidative action of the non-crystal form of UA because; oxidative stress plays a critical role in neurodegeneration including dopaminergic degeneration in PD [16,18]. As a natural antioxidant, UA provides up to 60% of the antioxidant capacity in human blood [19]. UA preserves the peroxidase activity of both cytosolic Superoxide Dismutase 1 (SOD and extracellular SOD3, which defend against the formation of superoxide (O^2−^) and peroxynitrite [20]. UA can also effectively prevent cytoskeleton from the insults caused by peroxynitrite-induced inactivation of cellular enzymes [21]. In addition, UA is capable of binding iron and inhibits iron-dependent ascorbate oxidation, thus preventing against oxidative stress-induced injuries. As such, a reduced UA concentration may adversely lead to increased oxidative stress and damage to neural cells. However, recent studies indicate that UA’s antioxidant property couldn’t explain all of its beneficial effects in the CNS. One study found that astroglia are required for UA to exert its protective effects on the spinal cord against injury [22]. UA stimulates expression of a glutamate transporter in astroglia, by which it protects neurons from glutamate-induced toxicity. Besides its capability of scavenging reactive oxygen species, UA can also indirectly confer neuronal protection via activation of astroglia. On the other hand, lower UA levels may be generated during CNS inflammation due to overconsumption of UA in scavenging excessive oxidative stress [23]. Notably, administration of UA to an experimental autoimmune/allergic encephalomyelitis mouse model, given either before or after the appearance of symptoms, promoted the survival rate of these mice [24]. Administration of UA effectively decreased oxidative stress and neuronal deaths in animal models of PD [25]. Interestingly, treatment of patients with a UA precursor, inosine, prevented progression of MS in all 11 patients tested and even improved the symptoms of some patients [26]. Thus, it is highly likely that a reduced serum UA level is a predictor rather than a consequence of neurodegeneration. Therefore, it is plausible to speculate that the neurodegenerative processes in patients with the neurological disorders may be exacerbated by the compromised neuroprotective effects due to the decreased UA levels.

More recent studies highlight the pathogenic property of UA that induces an inflammatory response contributing to gout and possibly UA-related CNS inflammatory diseases. Blocking antibodies against CD16 and CD11b selectively inhibit the activation of neutrophils and monocytes induced by MSU [27], indicating CD16^+^ and CD11b^+^ cells as important mediators in MSU-induced inflammation. In addition, more studies have been focused on the relationship between pathogen-associated molecular Pattern Recognition Receptors (PRRs) and MSU. MSU induces the activation of Nucleotide-Binding Oligomerization Domain (NOD)-like receptor protein 3 (NLRP3), a type of PRRs, and leads to assembly of a protein complex termed inflammasome. The NALP3 inflammasome is composed of NLRP3, apoptosis-associated speck-like protein containing a caspase recruitment domain (ACS), and caspase-1, whose function is to activate proinflammatory caspase-1. It processes pre-caspase-1 precursor into its p20-p10 heterodimer activated form, which cleaves and activates the well-known proinflammatory cytokines IL-1β and IL-18. Moreover, amyloid β associated with AD was also found to activate NLRP3 inflammasome-caspase-1 pathway [28]. A pilot study of IL-1 inhibitor in treating patients with acute gouty arthritis indicates its rapid beneficial effects [29], suggesting a possible therapeutic potential by blocking this pathway.

A few hypothetical mechanisms have been proposed to explain how a solid structure such as MSU can activate, post-translationally, intracellular receptors like NLRP3. One model suggests that the NLRP3 inflammasome is able to sensitize ionic perturbations induced by MSU crystals, especially potassium efflux [30]. It remains undetermined whether MSU activates specific ion channels or, more likely, causes a non-selective membrane damage resulting in increase in ion permeability. A second model proposes that the generation of reactive oxygen species induced by MSU is essential for NLRP3 inflammation activation [31]. A third model claims that considering the size of crystalline, MSU is too large to be efficiently phagocytized, and thus induces destabilization of lysosomes, which leads to the release of cathepsin B. This process may be facilitated by NLRP3. Although evidence can be found to support each of these three models, much is unknown regarding the details delineating each step. It is possible that all the three models may work in parallel or in tandem subsequences for inflammasome activation [10].

Since MSU is a direct and potent inflammasome activator, it is conceivable to speculate that high levels of UA may induce CNS inflammation and contribute to neurological disorders as well. Although hypouricemia has been associated with several neurological diseases, it has also been found that high levels of UA, which activates the NLRP3 inflammasome, are upregulated in the cerebrospinal fluid of MS patients [32]. Thus, hyperuricemia may also contribute to neurodegenerative diseases by activating the NLRP3 inflammasome.

## Conclusion

In summary, hyperuricemia has long been well known with gout. More recently, hyperuricemia has also been postulated to play a role in neurological diseases. Emerging studies of the pathological mechanisms of gout, pseudogout, and its relationship with neurological diseases have provided insight into the underlying mechanisms of MSU in gout, calcium pyrophosphate dehydrate in pseudogout, and amyloid β in AD as induced NLRP3 inflammasome/caspase-1 activation-mediated inflammatory pathologies. MSU crystal is recognized by NLRP3, which triggers the release of active proinflammatory cytokine IL-1β through NLRP3 inflammasome activation. The pro-inflammatory role of hyperuricemia provides the rationale for targeting NLRP3 and IL-1β as a potential disease-modifying therapy for neurological disorders. More questions remain to be addressed and await answers. If clarified and confirmed, UA could be an important predictor and a potentially modifiable factor affecting the rate of progression in neurological degeneration.

## References

[1] Cascalheira JF, Joao SS, Pinhancos SS, Castro R, Palmeira M (2009). Serum homocysteine: interplay with other circulating and genetic factors in association to Alzheimer’s type dementia. Clin Biochem.

[2] Liu B, Shen Y, Xiao K, Tang Y, Cen L (2012). Serum uric acid levels in patients with multiple sclerosis: a meta-analysis. Neurol Res.

[3] Paganoni S, Zhang M, Quiroz Zarate A, Jaffa M, Yu H (2012). Uric acid levels predict survival in men with amyotrophic lateral sclerosis. J Neurol.

[4] Tabunoki H, Ono H, Ode H, Ishikawa K, Kawana N (2013). Identification of key uric acid synthesis pathway in a unique mutant silkworm Bombyx mori model of Parkinson’s disease. PLoS One.

[5] Reitz C, Brayne C, Mayeux R (2011). Epidemiology of Alzheimer disease. Nat Rev Neurol.

[6] Barker DJ, Osmond C, Golding J, Kuh D, Wadsworth ME (1989). Growth in utero, blood pressure in childhood and adult life, and mortality from cardiovascular disease. BMJ.

[7] Jones DP, Mahmoud H, Chesney RW (1995). Tumor lysis syndrome: pathogenesis and management. Pediatr Nephrol.

[8] Becker M, Roessler BJ (1995). Hyperuricemia and gout. The metabolic and Molecular Bases of Inherited Disease.

[9] Jin M, Yang F, Yang I, Yin Y, Luo JJ (2012). Uric acid, hyperuricemia and vascular diseases. Front Biosci (Landmark Ed).

[10] Yin Y, Pastrana JL, Li X, Huang X, Mallilankaraman K (2013). Inflammasomes: sensors of metabolic stresses for vascular inflammation. Front Biosci (Landmark Ed).

[11] Andreadou E, Nikolaou C, Gournaras F, Rentzos M, Boufidou F (2009). Serum uric acid levels in patients with Parkinson’s disease: their relationship to treatment and disease duration. Clin Neurol Neurosurg.

[12] Annanmaki T, Muuronen A, Murros K (2007). Low plasma uric acid level in Parkinson’s disease. Mov Disord.

[13] Auinger P, Kieburtz K, McDermott MP (2010). The relationship between uric acid levels and Huntington’s disease progression. Mov Disord.

[14] De Vera M, Rahman MM, Rankin J, Kopec J, Gao X (2008). Gout and the risk of Parkinson’s disease: a cohort study. Arthritis Rheum.

[15] Pan M, Gao H, Long L, Xu Y, Liu M (2013). Serum uric acid in patients with Parkinson’s disease and vascular parkinsonism: a cross-sectional study. Neuroimmunomodulation.

[16] Schlesinger I, Schlesinger N (2008). Uric acid in Parkinson’s disease. Mov Disord.

[17] Maesaka JK, Wolf-Klein G, Piccione JM, Ma CM (1993). Hypouricemia, abnormal renal tubular urate transport, and plasma natriuretic factor(s) in patients with Alzheimer’s disease. J Am Geriatr Soc.

[18] Jenner P (2003). Oxidative stress in Parkinson’s disease. Ann Neurol.

[19] Ames BN, Cathcart R, Schwiers E, Hochstein P (1981). Uric acid provides an antioxidant defense in humans against oxidant- and radical-caused aging and cancer: a hypothesis. Proc Natl Acad Sci U S A.

[20] Hink HU, Fukai T (2002). Extracellular superoxide dismutase, uric acid, and atherosclerosis. Cold Spring Harb Symp Quant Biol.

[21] Pacher P, Beckman JS, Liaudet L (2007). Nitric oxide and peroxynitrite in health and disease. Physiol Rev.

[22] Du Y, Chen CP, Tseng CY, Eisenberg Y, Firestein BL (2007). Astroglia-mediated effects of uric acid to protect spinal cord neurons from glutamate toxicity. Glia.

[23] Drulovic J, Dujmovic I, Stojsavljevic N, Mesaros S, Andjelkovic S (2001). Uric acid levels in sera from patients with multiple sclerosis. J Neurol.

[24] Hooper DC, Spitsin S, Kean RB, Champion JM, Dickson GM (1998). Uric acid, a natural scavenger of peroxynitrite, in experimental allergic encephalomyelitis and multiple sclerosis. Proc Natl Acad Sci U S A.

[25] Duan W, Ladenheim B, Cutler RG, Kruman II, Cadet JL (2002). Dietary folate deficiency and elevated homocysteine levels endanger dopaminergic neurons in models of Parkinson’s disease. J Neurochem.

[26] Spitsin S, Hooper DC, Leist T, Streletz LJ, Mikheeva T (2001). Inactivation of peroxynitrite in multiple sclerosis patients after oral administration of inosine may suggest possible approaches to therapy of the disease. Mult Scler.

[27] Barabe F, Gilbert C, Liao N, Bourgoin SG, Naccache PH (1998). Crystal-induced neutrophil activation VI. Involvment of FcgammaRIIIB (CD16) and CD11b in response to inflammatory microcrystals. FASEB J.

[28] Halle A, Hornung V, Petzold GC, Stewart CR, Monks BG (2008). The NALP3 inflammasome is involved in the innate immune response to amyloid-beta. Nat Immunol.

[29] So A, De Smedt T, Revaz S, Tschopp J (2007). A pilot study of IL-1 inhibition by anakinra in acute gout. Arthritis Res Ther.

[30] Martinon F, Mayor A, Tschopp J (2009). The inflammasomes: guardians of the body. Annu Rev Immunol.

[31] Li X, Fang P, Mai J, Choi ET, Wang H (2013). Targeting mitochondrial reactive oxygen species as novel therapy for inflammatory diseases and cancers. J Hematol Oncol.

[32] Amorini AM, Petzold A, Tavazzi B, Eikelenboom J, Keir G (2009). Increase of uric acid and purine compounds in biological fluids of multiple sclerosis patients. Clin Biochem.

